# Association between *p53* Pro72Arg polymorphism and prostate cancer risk: a meta-analysis

**DOI:** 10.1016/S1674-8301(11)60003-1

**Published:** 2011-01

**Authors:** Lifeng Zhang, Ning Shao, Qianqian Yu, Lixin Hua, Yuanyuan Mi, Ninghan Feng

**Affiliations:** a Department of Urology, the First Affiliated Hospital of Nanjing Medical University, Nanjing, Jiangsu 210029, China;; b Department of Ophthalmology, Wuxi People's Hospital Affiliated with Nanjing Medical University, Wuxi, Jiangsu 214023, China.

**Keywords:** p53, prostate cancer, polymorphism, meta-analysis, risk

## Abstract

The tumor suppressor gene *p53* appears to be important in the development of many human cancers, such as prostate cancer. The association of *p53* codon72 polymorphism with prostate cancer has been widely reported; however, the results are inconsistent. To derive a more precise estimation of this relationship, we performed an updated meta-analysis from 10 case-control studies. We conducted a search in the PubMed database without a language limitation, covering all papers published until July 2010. *Risk ratios (RR)* with 95% *confidence intervals* *(CIs)* were used to assess the strength of the association. Ten studies including 1,196 cases and 1,704 controls were selected. Overall, no significant differences of total prostate cancer risk and *p53* codon polymorphism was found (Pro/Pro *vs* Arg/Arg, *RR* = 1.12, 95%*CI*=0.74-1.70, *P*_heterogeneity_ = 0.016, *I*^2^ = 55.8%; Pro/Pro+Pro/Arg *vs* Arg/Arg, *RR* = 1.05, 95%*CI*=1.00-1.11, *P*_heterogeneity_ = 0.077, *I*^2^ = 51.1%). In the stratified analysis by ethnicity, the same results were found. However, in the control subgroup, there was a modest decreased association between prostate cancer risk and population-based control subjects under the recessive genetic model (RR = 0.31, 95%CI=0.10-0.91, *P*_heterogeneity_ = 0.110, *I*^2^ =60.8%). This meta-analysis suggested that *p53* codon Pro72Arg polymorphism could be weakly associated with prostate cancer risk.

## INTRODUCTION

Prostate cancer (PCa) is the most commonly seen male malignancy and the second leading cause of cancer deaths in men in the United States, with estimated 192,000 new cases and 27,000 deaths in 2009[1]. The cause of PCa is heterogeneous, possibly involving both genetic and environmental factors[2]. PCa as a cause of death by cancer varies remarkably according to tumor grade, stage, age, and ethnic or racial groups.

The p53 transcription factor is encoded by the *TP53* gene, which is located on chromosome 17q13[3] and is one of the most commonly mutated genes in all types of human cancer. The *p53* gene and its encoded protein play a central role in regulating cell cycle progression, DNA repair, cellular growth and apoptosis[4],[5]; thus, it can function as a tumor suppressor. Because p53 can suppuss tumor development, and control apoptosis and cell cycle checkpoint in cells under physiologicall stress, it is one of the most intensely studied human proteins and is often called the “guardian of the genome” [5].

A common variant, a single nucleotide polymorphism (SNP) at codon72 (from CGC to CCC in exon 4, rs1042522), alters activities of p53. The C to G change results in a proline (Pro) to arginine (Arg) amino acid substitution in the proline-rich region that is essential for p53-mediated apoptosis[6],[7]. The proline variant has increased transcriptional transactivation activities and appears to induce a higher level of G1 cell-cycle arrest; however, the arginine allele has been associated with induction of apoptosis and suppression of cellular transformation by binding more efficiently to the promoters of pro-apoptotic genes[8]–[11].

The association between *p53* gene codon72 polymorphism and tumor formation has been extensively studied, including in ovarian, lung, cervical, and colon cancer and PCa. Of the ten publications on PCa[12]–[21], some studies have found that *p53* codon Pro72 polymorphism was associated with a decreased risk of PCa, while others reported no association or an association between Arg and decreased risk of PCa. Therefore, whether the polymorphism of *p53* gene codon72 is associated with PCa or not is still controversial.

Taking into consideration the extensive role of *p53* codon72 in PCa, and to derive a more precise estimation of the association of Pro72Arg polymorphism in *p53* and PCa, we performed a meta-analysis of all eligible case-control studies.

## MATERIALS AND METHODS

### Literature search

We conducted searches on the PubMed database (http://www.ncbi.nlm.nih.gov/), last search updated on July 2010, with the keywords “p53” or “TP53”, “polymorphism” and “prostate cancer” or “prostate”. Using these terms, a total of 101 articles were retrieved, of which 10 articles[12]–[21] met the inclusion criteria indicated below on studies examining the association between *p53* codon Pro72Arg polymorphism and PCa risk.

### Inclusion and exclusion criteria

Studies testing the association between *p53* codon Pro72Arg polymorphism and PCa were considered if all the following inclusion criteria were met: 1) the study assessed the correlation between global cancer and at least one of the polymorphisms cited above; 2) case-control studies; 3) control subjects were matched with case patients in age and gender; 4) only full-text manuscripts were included. Major exclusion criteria were: 1) no control population; 2) no available genotype frequency; 3) duplication of previous publications; 4) manuscripts with a clear bias of accrual.

### Data extraction

Two of the authors reviewed the results of each of the database searches to make sure that all published papers were not missed. Data were collected based on the first author's last name, year of publication, country of origin, ethnicity, cancer type, sample size (cases/controls), genotyping methods, age range in cases and controls, source of control and Hardy–Weinberg equilibrium (HWE) of controls.

### Genotyping methods

Genotyping for SNP of *p53* codon Pro72Arg gene was conducted using polymerase chain reaction-restriction fragment length polymorphism (PCR-RFLP)[12]–[16],[19]–[21] and PCR-sequencing[17],[18].

### Statistical analysis

Crude risk ratios (RR) with 95% confidence intervals (CIs) were used to measure the strength of the association between *p53* codon Pro72Arg polymorphism and PCa based on the genotype frequencies in cases and controls. Subgroup analysis stratified by ethnicity was performed first. Ethnicity was categorized as Caucasian, Asian and African. Source of control subgroup analysis was performed on two classifications: population-based and hospital-based.

The fixed effects model and the random effects model were used to calculate the pooled RR. The statistical significance of the summary RR was determined by *Z* test. Heterogeneity assumption was evaluated with a chi-square-based *q* test among the studies. A *P* value of more than 0.05 for the *q*-test indicated a lack of heterogeneity among the studies. In order to better evaluate the extent of heterogeneity between studies, the *I*^2^ test was also used. As a guide, *I*^2^ values of <25% may be considered ‘low’, value of ∼50% may be considered ‘moderate’ and values of >75% may be considered ‘high’[22]. If *P*≤0.05, or I^2^≥50%, a random-effects model using the DerSimonian–Laird method[23], which yields wider confidence intervals, was adopted; otherwise if *P* > 0.05, and I^2^<50%, a fixed-effects model using the Mantel–Haenszel method[24] was used. For *p53* codon Pro72Arg, we investigated the association between genetic variants and PCa risk in allelic contrast (Pro-allele *vs* Arg-allele), homozygote comparison (Pro/Pro *vs* Arg/Arg), heterozygote comparison (Pro/Arg *vs* Arg/Arg), dominant genetic model (Pro/Pro+Pro/Arg *vs* Arg/Arg) and recessive genetic model (Pro/Pro *vs* Pro/Arg+Arg/Arg). The funnel plot asymmetry was assessed with Egger's test. Publication bias was assessed with Egger's test; *P* < 0.05 was considered statistically significant[25]. The departure of frequencies of *p53* codon Pro72Arg polymorphism from expectation under HWE was assessed by *X*^2^ test in controls using the Pearson chi-square test for goodness of fit, *P* < 0.05 was considered significant. All statistical tests for this meta-analysis were performed with STATA software (Version 10.0, StataCorp LP, College Station, TX, USA).

## RESULTS

### Eligible studies

Of the 101 abstracts retrieved through the search criteria, 71 were irrelevant, six articles were reviews, 13 studies were excluded because they did not concern *p53* codon Pro72Arg, and one study[26] was excluded as it did not report the relevant genotype frequencies. As a result, 10 case-control articles were included in our meta-analysis[12]–[21].

The characteristics of the eligible studies are presented in ***Table 1*** and ***Table 2***. The genetic distribution of the control groups of eight studies was consistent with HWE[12]–[17],[19]–[20], while the remaining two studies[18],[21] were not. In one study[15], 89 Caucasian men were studied, of whom 41 cases had a diagnosis of PCa and the remaining 48 controls had a diagnosis of benign prostatic hyperplasia (BPH). However, in another study[16], a total of 200 patients with PCa, 181 with BPH, and 247 male controls were included, we used the 247 male as controls but not the 181 with BPH.

**Table 1:** Characteristics of studies of *p53* codon Pro72Arg polymorphism included in this meta-analysis

First author	Country	Ethnicity	Cases/Controls	Age range (year)		Source of control	Genotyping methods
				Cases	Controls		
Ricks-Santi[12]	USA	African	245/178	41-95(65.58±NA)	35-89(57.36±NA)	HB	PCR-RFLP
Hirata[13]	Japan	Asian	167/167	NA(68±10)	NA(68±10)	HB	PCR-RFLP
Quiñones[14]	Chile	Caucasian	60/117	NA(60.7±12.85)	NA(60.36 ±14.25)	HB	PCR-RFLP
Leiros[15]	Argentina	Caucasian	41/48	NA(>60±NA)	NA(>60±NA)	PB	PCR-RFLP
Huang[16]	China(Taiwan)	Asian	200/247	NA(72.2±7.7)	NA(72.4±6.5)	HB	PCR-RFLP
Wu[17]	China(Taiwan)	Asian	92/126	49-96(70.6 ±8.97)	60-87(66.5±5.08)	HB	PCR-sequencing
Henner[18]	USA	Caucasian	109/146	44-86(67±10)	24-79(52±11)	PB	PCR-sequencing
Hirata[19]	Japan	Asian	140/167	NA(68±10)	NA(68±10)	HB	PCR-RFLP
Wu[20]	Japan	Asian	28/403	NA(66.9±7.5)	NA(35.1±16)	HB	PCR-RFLP
Suzuki[21]	Japan	Asian	114/105	40-88(70.3±7.7)	51-88(71.2±7.0)	HB	PCR-RFLP

HB: hospital-based control; PB: population-based control; PCR-RFLP: polymerase chain reaction-restriction fragment length polymorphism; NA: not available.

(mean±SD)

**Table 2:** Distribution of *p53* codon Pro72Arg genotype among PCa cases and controls included in the meta-analysis

First author	Cases			Controls				Frequency of Pro allele
	Pro/Pro	Pro/Arg	Arg/Arg	Pro/Pro	Pro/Arg	Arg/Arg	*P*_HWE_	
Ricks-Santi[12]	73	135	37	70	86	22	0.575	63.48
Hirata[13]	22	89	56	26	80	61	0.978	39.52
Quiñones[14]	14	24	22	13	45	59	0.330	30.34
Leiros[15]	2	17	20	2	23	23	0.199	28.13
Huang[16]	42	92	66	54	109	84	0.102	43.93
Wu[17]	20	61	11	30	53	43	0.093	44.84
Henner[18]	2	41	66	15	38	93	0.001	23.29
Hirata[19]	20	75	45	26	80	61	0.978	39.52
Wu[20]	2	14	12	44	189	170	0.427	34.37
Suzuki[21]	20	46	48	7	57	41	0.029	33.81

HB: hospital-based control; PB: population-based control; PCR-RFLP: polymerase chain reaction-restriction fragment length polymorphism; NA: not available.

### Test of heterogeneity

As shown in ***Table 3***, there was significant heterogeneity for homozygote comparison (*P*_heterogeneity_ = 0.016), recessive genetic model (*P*_heterogeneity_ =0.018) and heterozygote comparison (*P*_heterogeneity_ = 0.035), but not for the allelic contrast (*P*_heterogeneity_ = 0.084) and the dominant model (*P*_heterogeneity_ = 0.077), because the *P* values were more than 0.05 for *Q*-tests. However, subgroup analysis regarding ethnicity and source of control were conducted, and the *P* value for heterogeneity indicated a reduced or absent heterogeneity.

**Table 3:** Stratified analyses of the *p53* codon Pro72Arg polymorphism and PCa risk

Genetic model (No.of studies: Cases/Controls)	Main effects of p53 codon Pro72Arg polymorphism in PCa				
	*RR(95%CI)*	*P*_heterogeneity_	*P*	*I*^2^ (%)	Analysis model
Total(10:l,196/1,704)					
Allelic contrast	1.02(0.96-1.09)	0.084	0.532	41.1	fixed effects model
Homozygote comparison	1.12(0.74-1.70)	0.016	0.590	55.8	random effects model
Heterozygote comparison	1.22(0.94-1.60)	0.035	0.136	50.1	random effects model
Dominant genetic model	1.05(1.00-1.11)	0.077	0.069	51.1	fixed effects model
Recessive genetic model	0.96(0.67-1.37)	0.018	0.815	55.1	random effects model
Ethnicity					
Asian (6:741/1,215)					
Allelic contrast	1.05(0.97-1.14)	0.601	0.228	0.0	fixed effects model
Homozygote comparison	1.13(0.93-1.37)	0.229	0.227	27.5	fixed effects model
Heterozygote comparison	1.27(0.85-1.91)	0.008	0.248	68.3	random effects model
Dominant genetic model	1.24(0.89-1.73)	0.039	0.212	57.3	random effects model
Recessive genetic model	1.00(0.80-1.24)	0.270	0.992	21.8	fixed effects model
Caucasian (3,210/311)					
Allelic contrast	1.10(0.90-1.34)	0.073	0.346	61.7	fixed effects model
Homozygote comparison	0.90(0.14-5.69)	0.007	0.911	79.6	random effects model
Heterozygote comparison	1.18(0.95-1.48)	0.458	0.139	0.0	fixed effects model
Dominant genetic model	1.13(0.93-1.36)	0.435	0.214	0.0	fixed effects model
Recessive genetic model	0.83(0.13-5.12)	0.006	0.838	80.3	random effects model
African (1:245/178)					
Allelic contrast	0.77(0.58-1.02)	-	0.072	-	random effects model
Homozygote comparison	0.62(0.33-1.15)	-	0.132	-	random effects model
Heterozygote comparison	0.93(0.52-1.69)	-	0.820	-	random effects model
Dominant genetic model	0.79(0.45-1.40)	-	0.422	-	random effects model
Recessive genetic model	0.65(0.44-0.98)	-	0.041	-	random effects model
Source of control					
Hospital-based (4:1,046/1,510)					
Allelic contrast	1.03(0.97-1.10)	0.060	0.369	52.2	fixed effects model
Homozygote comparison	1.24(0.83-1.85)	0.037	0.298	53.2	random effects model
Heterozygote comparison	1.23(0.90-1.69)	0.020	0.199	58.1	random effects model
Dominant genetic model	1.21(0.91-1.61)	0.035	0.181	53.6	random effects model
Recessive genetic model	1.02(0.73-1.43)	0.038	0.896	52.8	random effects model
Population-based (2:150/194)					
Allelic contrast	0.91(0.69-1.19)	0.798	0.488	0.0	fixed effects model
Homozygote comparison	0.34(0.11-1.01)	0.157	0.053	50.1	fixed effects model
Heterozygote comparison	1.17(0.88-1.55)	0.217	0.276	34.5	fixed effects model
Dominant genetic model	1.04(0.80-1.34)	0.575	0.781	0.0	fixed effects model
Recessive genetic model	0.31(0.10-0.91)	0.110	0.032	60.8	fixed effects model

HB: hospital-based of control; PB: population-based of control; PCR-RFLP: polymerase chain reaction-restriction fragment length polymorphism; NA: not available.

### Meta-analysis results

**Fig. 1:**
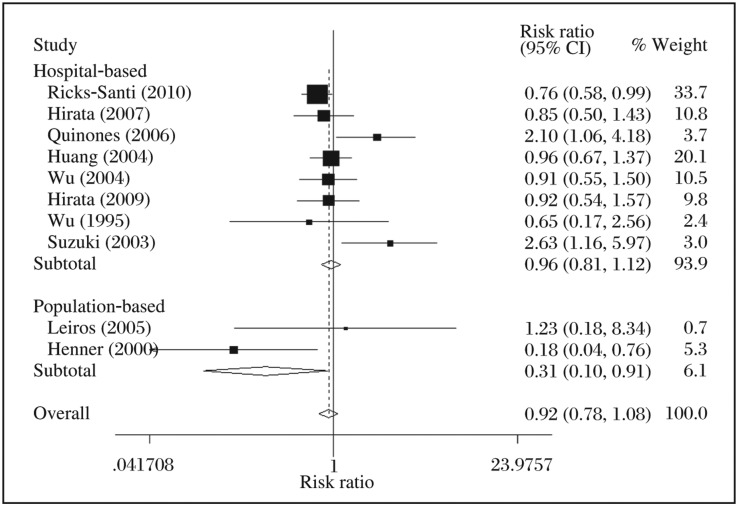
Forest plot of prostate cancer risk associated with the *p53* codon Pro72Arg polymorphism by source of control in recessive genetic mode. The squares and horizontal lines correspond to the study-specific relative risk (*RR*) and 95% confidence interval (*CI*). The area of the squares reflects the weight (inverse of the variance). The diamond represents the summary *RR* and 95% *CI*.

Regarding *p53* codon Pro72Arg, the results of the meta-analysis are presented in detail in ***Table 3***. No statistically significant association was detected in overall PCa risk [allelic contrast, *RR* = 1.02, 95%*CI* (0.96-1.09), *P* = 0.532, *I*^2^ = 41.1%; homozygote comparison, *RR* = 1.12, 95%*CI* (0.74-1.70), *P* = 0.590, *I*^2^ = 55.8%; heterozygote comparison, *RR* = 1.22, 95%*CI* (0.94-1.60), *P* = 0.136, *I*^2^ = 50.1%; dominant models, *RR* = 1.05, 95%*CI* (1.00-1.11), *P* = 0.069, *I*^2^ = 51.1% and recessive genetic model, *RR* = 0.96, 95%*CI* (0.67-1.37), *P* = 0.815, *I*^2^ = 55.1%]. Additionally, in the stratified analysis by ethnicity, no significant association between PCa and *p53* codon72 polymorphism was found in each of the three ethnicities. However, in the subgroup of source of control, we found a significantly decreased risk for PCa in population-based control subjects (recessive genetic model, *RR* = 0.31, 95%*CI*=0.10-0.91, *P*_heterogeneity_ = 0.110, *I*^2^ =60.8%, ***Fig. 1***).

The distribution of genotypes in the controls of two studies[18],[21] was not consistent with the HWE, when they were excluded, significant heterogeneity did not change and the positive association still existed. The data indicated that *p53* codon72 polymorphism has little association with the development of PCa.

### Sensitivity analysis

We use one-way sensitivity analysis[27] to determine whether modification of the inclusion criteria of the meta-analysis affected the final results. These were carried out by limiting the meta-analysis to the studies conforming to HWE and altering corresponding statistical variables and analysis models. Moreover, no other single study influenced the summary RR qualitatively as indicated by sensitivity analysis. Hence, the results of the sensitivity analysis suggest that the data in this meta-analysis are relatively stable and credible.

### Bias diagnosis

**Fig. 2:**
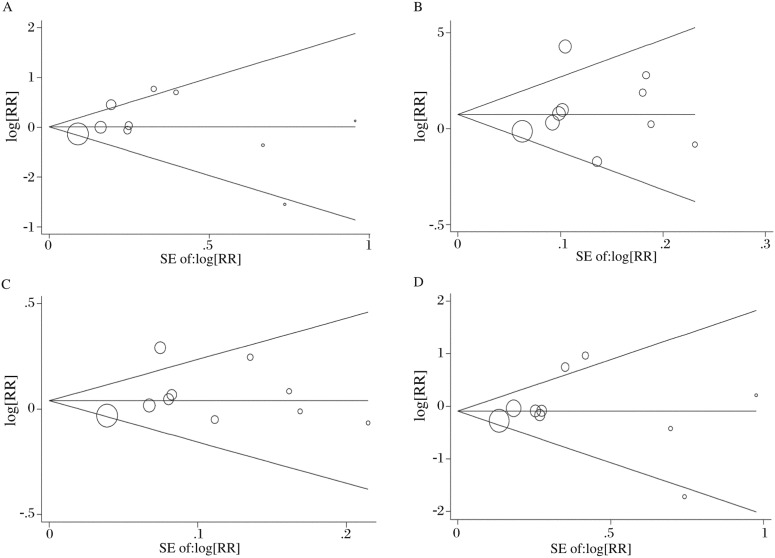
Funnel plot analysis to detect publication bias. Each point represents a separate study for the indicated association. A: homozygote comparison; B: heterozygote comparison; C: dominant genetic model; D: recessive genetic model. CI: confidence interval; RR: relative risk; SE: standard error.

The Begg's funnel plot and Egger's test were performed to assess the publication bias of the literature. The shape of the funnel plots did not reveal any evidence of obvious asymmetry in all five models. Then, Egger's test was used to provide statistical evidence of funnel plot symmetry. The results still did not suggest any evidence of publication bias (allelic contrast, *t* = 1.13, *P* = 0.292; homozygote comparison, *t* = 0.07, *P* = 0.958; heterozygote comparison, *t* = 0.63, *P* = 0.549; dominant model, *t* = 0.85, *P* = 0.418; recessive genetic model, *t* = 0.37, *P* = 0.719; ***Table 4*** and ***Fig. 2***).

**Table 4:** Publication bias tests (Begg's funnel plot for publication bias test) for *p53* codon Pro72Arg polymorphism

Genetic type	Coefficient	Standard error	*t*	*P* value	95%CI of intercept
Allelic contrast	1.151	1.020	1.13	0.292	(-1.201, 3.502)
Homozygote comparison	0.095	1.410	0.07	0.958	(-3.157, 3.348)
Heterozygote comparison	1.330	2.124	0.63	0.549	(-3.567, 6.227)
Dominant genetic model	0.836	0.980	0.85	0.418	(-1.424, 3.096)
Recessive genetic model	0.445	1.194	0.37	0.719	(-2.310, 3.198)

CI: confidence interval; HB: hospital-based control; PB: population-based control; PCR-RFLP: polymerase chain reaction-restriction fragment length polymorphism; NA: not available.

## DISCUSSION

The *p53* gene, with a central role in tumor suppression by initiating apoptosis or inducing cell arrest at the G1/S-phase in response to DNA damage[4],[5], appears to play a prominent role in the pathogenesis of many kinds of cancer. Approximately 20% to 50% of prostatic carcinomas possess mutations of the *p53* gene[28]. The present meta-analysis included 1,196 cases and 1,704 controls concerning codon72 polymorphism in the promoter region of the *p53* gene. We also explored the association between the potentially functional polymorphism of *p53* and PCa risk.

Previously, relationships between the *p53* codon72 polymorphism and clinical parameters of diseases have been analyzed in several types of cancers. For example, Koushik *et al*.[29] reported that 72Arg genotype increased susceptibility to both cervical squamous cell carcinoma and adenocarcinoma, while three other studies recently[30]–[32] indicated that the Pro72 variant might increase the susceptibility to bladder cancer, esophageal squamous cell carcinoma and lung cancer in Asians, respectively. Moreover, non-associations of *p53* codon72 polymorphism with oral carcinoma[33], colorectal cancer[34] and breast cancer[35]–[36] risk were observed by meta-analysis. Some factors can influence this discrepancy. First, *p53* codon Pro72Arg polymorphism might play different roles in different cancers. Second, cancer is a multifactorial disease that results from complex interactions between many genetic and environmental factors. This means that there will not be a single gene or single environmental factor that has large effects on cancer susceptibility[37]. Environmental factors (e.g. smoking, dietary factors) add to the carcinogenic load to which humans are exposed, but exact numbers for added risk are generally less well established. Hence, *p53* codon72 polymorphism contribution to the susceptibility to cancer risk varies in different types of cancers.

Functional studies of the *p53* codon72 polymorphism have demonstrated different biological properties between the Arg and Pro alleles: the Pro variant can increase transcriptional transactivation, but the Arg allele has been associated with induction of apoptosis and suppression of cellular transformation by binding more efficiently to the promoters of pro-apoptotic genes[8]–[11]. In addition, the Arg allele enhances mutant *p53* binding to *p73*[38]. The half-lives of both polymorphic isoforms of *p53* are similar in normal phytohemagglutinin-stimulated lymphocytes, while the Pro isoform is twice as stable as the Arg isoform in Daudi cells[39]. These different functions can be explained by the different results of some publications in our meta-analysis. For example, Henner *et al*.[18] and Ricks-Santi *et al*.[12] suggested that men with the *p53* codon Pro72 genotype appeared to be at reduced risk of PCa, while Suzuki *et al*.[21] reported that the Pro/Pro genotype of *p53* codon72 was associated with a risk of PCa only in patients with a family history.

To the best of our knowledge, our results indicated that no significant association was found between *p53* codon Pro72Arg polymorphism and overall PCa risk. In the stratified analysis by ethnicity, the same results were found in all genotype models, while we only found that *p53* codon Pro72 polymorphism may weakly protect against PCa in population-based control subjects.

In our meta-analysis, the source of control subgroup analysis was performed on two classifications: population-based and hospital-based. In our searched publications, if the source of control was performed on population-based control subjects, source of case was also population-based; the same was true for as hospital-based cases and controls. We know that population-based data is better to represent the general causes of various types of disease (such as PCa) than hospital-based. In our results, we found a significantly decreased risk for PCa in population-based control subjects, although the number was quite small and just included two case-control studies. As a result, if the Pro to Arg amino acid alteration occured in the proline-rich region essential for p53-mediated apoptosis, the incidence of PCa would be expected to go down. This maybe helpful for finding the etiology of PCa.

Several limitations in this meta-analysis should be mentioned. First of all, the number of published studies included in our meta-analysis was not sufficiently large for a comprehensive analysis, particularly for any given ethnicity (especially African) site. Second, publication bias might have occurred and our Egger's test results may have a substantial risk of being affected by such bias, although the funnel plots as well as Egger's linear regression tests indicated no remarkable publication biases in the meta-analyses. Third, the interactions between gene–gene, gene–environment and even different polymorphic loci of the same gene may modulate PCa risk. Fourth, in some *p53* codon Pro72Arg polymorphism studies[14]–[15],[20], a small number of cases and/or controls were included. Fifth, our meta-analysis was based on unadjusted estimates. A more precise analysis should be conducted of individual information including other covariates such as age, sex and metastasis/differentiation status. Furthermore, the genetic distributions of the controls in the two studies[18],[21] were deviated from HWE, resulting in some inevitable biases. In spite of these constraints, our pooled analysis also had two advantages. First, a substantial number of cases and controls were pooled from different studies, which significantly increased the statistical power of the analysis. Second, the quality of case-control studies included in the current pooled analysis was satisfactory based on our selection criteria. Third, we did not detect any publication bias in ***Table 4***, suggesting that the results are relatively stable and the publication biases may not have had an obvious influence on the results of the meta-analysis.

In summary, our meta-analysis showed the evidence that the *p53* codon Pro72Arg polymorphism was associated with decreased PCa risk in population-based subjects in recessive genetic model. However, no significant association was found in any genetic model in the whole population and ethnic group. Therefore, further well designed large studies, particularly referring to gene-gene and gene-environment interactions, are warranted. These future studies should lead to better and comprehensive understanding of the association between the *p53* codon Pro72Arg polymorphism and PCa risk.

## References

[1] Jemal A, Siegel R, Ward E, Hao Y, Xu J, Thun MJ (2009). Cancer statistics, 2009. CA Cancer J Clin.

[2] Coughlin SS, Hall IJ (2002). A review of genetic polymorphisms and prostate cancer risk. Ann Epidemiol.

[3] Jin S, Levine AJ (2001). The p53 functional circuit. J Cell Sci.

[4] Lane DP (1992). Cancer. p53, guardian of the genome. Nature.

[5] Levine AJ (1997). p53, the cellular gatekeeper for growth and division. Cell.

[6] Sullivan A, Syed N, Gasco M, Bergamaschi D, Trigiante G, Attard M (2004). Polymorphism in wild-type p53 modulates response to chemotherapy in vitro and in vivo. Oncogene.

[7] Sakamuro D, Sabbatini P, White E, Prendergast GC (1997). The polyproline region of *p53* is required to activate apoptosis but not growth arrest. Oncogene.

[8] Whibley C, Pharoah PD, Hollstein M (2009). p53 polymorphisms: cancer implications. Nat Rev Cancer.

[9] Dumont P, Leu JI, Della Pietra AC, George DL, Murphy M (2003). The codon 72 polymorphic variants of p53 have markedly different apoptotic potential. Nat Genet.

[10] Leu JI, Dumont P, Hafey M, Murphy ME, George DL (2004). Mitochondrial *p53* activates Bak and causes disruption of a Bak-Mcl1 complex. Nat Cell Biol.

[11] Chipuk JE, Kuwana T, Bouchier-Hayes L, Droin NM, Newmeyer DD, Schuler M (2004). Direct activation of Bax by p53 mediates mitochondrial membrane permeabilization and apoptosis. Science.

[12] Ricks-Santi L, Mason T, Apprey V, Ahaghotu C, McLauchlin A, Josey D (2010). p53 Pro72Arg polymorphism and prostate cancer in men of African descent. Prostate.

[13] Hirata H, Hinoda Y, Kikuno N, Kawamoto K, Dahiya AV, Suehiro Y (2007). CXCL12 G801A polymorphism is a risk factor for sporadic prostate cancer susceptibility. Clin Cancer Res.

[14] Quiñones LA, Irarrázabal CE, Rojas CR, Orellana CE, Acevedo C, Huidobro C (2006). Joint effect among *p53*, CYP1A1, GSTM1 polymorphism combinations and smoking on prostate cancer risk: an exploratory genotype-environment interaction study. Asian J Androl.

[15] Leiros GJ, Galliano SR, Sember ME, Kahn T, Schwarz E, Eiguchi K (2005). Detection of human papillomavirus DNA and *p53* codon 72 polymorphism in prostate carcinomas of patients from Argentina. BMC Urol.

[16] Huang SP, Wu WJ, Chang WS, Wu MT, Chen YY, Chen YJ (2004). *p53* Codon 72 and p21 codon 31 polymorphisms in prostate cancer. Cancer Epidemiol Biomarkers Prev.

[17] Wu HC, Chang CH, Chen HY, Tsai FJ, Tsai JJ, Chen WC (2004). *p53* gene codon 72 polymorphism but not tumor necrosis factor-alpha gene is associated with prostate cancer. Urol Int.

[18] Henner WD, Evans AJ, Hough KM, Harris EL, Lowe BA, Beer TM (2001). Association of codon 72 polymorphism of p53 with lower prostate cancer risk. Prostate.

[19] Hirata H, Hinoda Y, Kikuno N, Suehiro Y, Shahryari V, Ahmad AE (2009). Bcl2 -938C/A polymorphism carries increased risk of biochemical recurrence after radical prostatectomy. J Urol.

[20] Wu WJ, Kakehi Y, Habuchi T, Kinoshita H, Ogawa O, Terachi T (1995). Allelic frequency of *p53* gene codon 72 polymorphism in urologic cancers. Jpn J Cancer Res.

[21] Suzuki K, Matsui H, Ohtake N, Nakata S, Takei T, Nakazato H (2003). A *p53* codon 72 polymorphism associated with prostate cancer development and progression in Japanese. J Biomed Sci.

[22] Higgins JP, Thompson SG, Deeks JJ, Altman DG (2003). Measuring inconsistency in meta-analyses. BMJ.

[23] DerSimonian R, Laird N (1986). Meta-analysis in clinical trials. Control Clin Trials.

[24] Mantel N, Haenszel W (1959). Statistical aspects of the analysis of data from retrospective studies of disease. J Natl Cancer Inst.

[25] Egger M, Davey Smith G, Schneider M, Minder C (1997). Bias in meta-analysis detected by a simple, graphical test. BMJ.

[26] Huang SP, Huang CY, Wang JS, Liu CC, Pu YS, Yu HJ (2007). Prognostic significance of *p53* and X-ray repair cross-complementing group 1 polymorphisms on prostate-specific antigen recurrence in prostate cancer post radical prostatectomy. Clin Cancer Res.

[27] Simmonds MC, Higgins JP, Stewart LA, Tierney JF, Clarke MJ, Thompson SG (2005). Meta-analysis of individual patient data from randomized trials: a review of methods used in practice. Clin Trials.

[28] Bookstein R (1994). Tumor suppressor genes in prostatic oncogenesis. J Cell Biochem Suppl.

[29] Koushik A, Platt RW, Franco EL (2004). *p53* codon 72 polymorphism and cervical neoplasia: a meta-analysis review. Cancer Epidemiol Biomarkers Prev.

[30] Jiang DK, Ren WH, Yao L, Wang WZ, Peng B, Yu L (2010). Meta-Analysis of Association Between TP53 Arg72Pro Polymorphism and Bladder Cancer Risk. Urology.

[31] Wang B, Wang D, Zhang D, Li A, Liu D, Liu H (2010). Pro variant of TP53 Arg72Pro contributes to esophageal squamous cell carcinoma risk: evidence from a meta-analysis. Eur J Cancer Prev.

[32] Yan L, Zhang D, Chen C, Mao Y, Xie Y, Li Y (2009). TP53 Arg72Pro polymorphism and lung cancer risk: a meta-analysis. Int J Cancer.

[33] Zhuo XL, Li Q, Zhou Y, Cai L, Xiang ZL, Yuan W (2009). Study on TP53 codon 72 polymorphisms with oral carcinoma susceptibility. Arch Med Res.

[34] Dahabreh IJ, Linardou H, Bouzika P, Varvarigou V, Murray S (2010). TP53 Arg72Pro polymorphism and colorectal cancer risk: a systematic review and meta-analysis. Cancer Epidemiol Biomarkers Prev.

[35] Ma Y, Yang J, Liu Z, Zhang P, Yang Z, Wang Y (2010). No significant association between the TP53 codon 72 polymorphism and breast cancer risk: a meta-analysis of 21 studies involving 24,063 subjects. Breast Cancer Res Treat.

[36] Hu Z, Li X, Yuan R, Ring BZ, Su L (2010). Three common TP53 polymorphisms in susceptibility to breast cancer, evidence from meta-analysis. Breast Cancer Res Treat.

[37] Pharoah PD, Dunning AM, Ponder BA, Easton DF (2004). Association studies for finding cancer-susceptibility genetic variants. Nat Rev Cancer.

[38] Marin MC, Jost CA, Brooks LA, Irwin MS, O'Nions J, Tidy JA (2000). A common polymorphism acts as an intragenic modifier of mutant *p53* behaviour. Nat Genet.

[39] Zhang W, Hu G, Deisseroth A (1992). Polymorphism at codon 72 of the *p53* gene in human acute myelogenous leukemia. Gene.

